# Essay on the Natural History, Origin, Composition, &c. Of Mineral Waters

**Published:** 1833-07-01

**Authors:** 


**Essay on the Natural History, Origin, Composition, and Medicinal Effects of Mineral and Thermal Springs. By *Meredith Gairdner*, M.D. 12mo, pp. 420. Edinburgh.**

The work is divided into four great sections, illustrative of the composition, the geographical and geognostical situation, the origin, and, lastly, the medical uses of mineral waters. The chemical history is given at great length and with much ability; reference being made to all the best books on the subject. In the early ages of analysis, it was believed that the native fountains of the earth contained almost every mineral substance; and especially the noble metals, as gold, mercury, copper, and arsenic;—hence the ready inference of their wonder-working cures. Hoffman was the first to reject, at at least in part, this merely whimsical idea, substituting one of his own nearly as gratuitous. "Scaturigines delectu quasi instituta ea tantum quæ partibus corporis nostri tam solidis quam fluidis accommoda et amicissima sunt, e terræ visceribus elambunt et in societatem suam admittant."

The labours of Black, Lavoisier, and Berthollet, having introduced into the schools of medicine a more rigid and searching chemistry, many of the supposed active ingredients of native waters were found wanting, and the predominating salts, those on which the effective virtues of the spring depended, discovered to be those which are most ordinarily used in diet or in medicine, such as the carbonate, muriate, and sulphate of soda, muriate of magnesia, and so forth. But it is in chemistry, as in most other pursuits of the human mind; credulity is often followed by scepticism and infidelity. We mean not to disparage the labours of those eminent men who were chiefly instrumental in bringing in the science of chemical analysis—they were quite right, in refusing to admit the existence of any substance in mineral waters, if this existence was not proved by the test of demonstration;—for this is the key to the philosophy of Bacon: but it is interesting to observe the revolution of events; for now it is ascertained that not a few of the conjectural ingredients of Paracelsus and his followers are truly and really present;—we owe this discovery to the recent labours of Berzelius, Bischof, Daubeny, Brandes, &c. In 1822 the first of these eminent analysts detected in the hot springs of Karlsbad no fewer than six new salts, in addition to those previously known.

As we might naturally expect, the most generally diffused and most predominating ingredients are the alkaline and earthy salts; and of these, such as have for their bases soda, lime, magnesia, and alumina, all substances which enter largely into the composition of the solid crust of the globe, are much more prevalent than those of potassa, ammonia, baryta, &c. Lithion has been hitherto found only in very few springs, as those of Ems, Pyrmont, Karlsbad, and Kreuznach. The acids which are the most generally and largely diffused are the carbonic, muriatic, and sulphuric; they are, as a matter of course, in union with the alkaline and earthy bases; and thus are generated the various neutral salts: those hitherto discovered in mineral waters are—the sulphates of soda, magnesia, lime, alumina, potash, baryta, and of lithion;—the muriates of soda, magnesia, lime, potash, alumina, ammonia, and of lithion;—the carbonates of soda, lime, magnesia, alumina, potash, and of lithion;—the phosphates of soda, potash, alumina and lime;—the fluate of lime—the borate of soda—and the nitrates of potash and of lime. The three last classes of neutral salts are very rare, and have been hitherto detected only in minute quantities. The discovery of the salts of lithion has been made by Berzelius in the waters of Karlsbad, Franzenbrunn, Marienbad; by John, at Gleissen in Brandenburg; and in a few of the Pyrmont springs by Brandes and Krüger. It is probable that they exert little influence on the medicinal properties of the waters; yet the fact of their presence is most interesting, from their rarity, even in the mineral crust of the globe; whether they are more largely distributed in the treasures of the bowels of the earth, is a problem for future inquirers. The number of metallic salts which have been found in springs is small, and their quantity is generally very limited. Iron is by far the most abundantly diffused, and, indeed, scarcely any mineral water is destitute of it. Manganese has been found in the Karlsbad Sprudel, and the waters of Saidschutz and Kreuznach—zinc in the mineral water of Ronneby, in Sweden, and copper, although it has not hitherto been detected in any true spring, exists freely in the pools of copper-mines, of Glamorganshire, in Wales, Fahlun, in Sweden, and Neusohl in Hungary. Silica, from its very sparing solubility, seldom exists in any quantity, except when mechanically suspended; in a state of minute division, it is found in a great number of cold springs, mineral as well as common, and it is a curious and interesting fact, that it is much more prevalent and more abundant in the springs of low, calcareous, and sandstone districts, than in those waters which flow over rocky and siliceous channels, in mountainous regions. Chemically dissolved, it is found only in thermal springs, or in cold ones, which contain a large quantity of soda, or of carbonic acid in excess. By far the most striking examples are the boiling springs of Geyser and Rycum, in Iceland, where it amounts to one-half of all the solid constituents, the former containing 5.40, the latter 3.73. Iodine was discovered by Balard in the waters of the Mediterranean, and by Pfaff in those of the Baltic. In 1820, M. Angelina detected it in the saline waters of Sales, in Piedmont, which contain one-twelfth of muriate of soda, and where it was till then unknown, although these waters had been repeatedly before carefully analysed. It always exists naturally in the form of a hydriodate, generally of soda, more rarely of potass. It has since been found in a great many springs, as in those of Genesio and Castel Nuovo—of Bonnington, near Edinburgh—of Leamington, Cheltenham—of Saltzhausen, near Frankfort, of Kreuznach, Heilbrunn, and Halle.

"In South America, Boussingault has found traces of the hydriodate of magnesia in the saline waters of Antioquia in Peru; and Dr. Mill mentions the occurrence of hydriodate of potass in a spring 16 leagues from Popayan, at an altitude of 10,000 feet above the level of the sea, and between 80 and 90 miles distant from it in a straight line.

I may just recal to the recollection of the reader, that not a trace of iodine has yet been found in rock-salt." 28.

The quantity of this very active substance is always exceedingly minute;

Dr. Daubeny never detected more than one grain in ten gallons of any mineral water; this was in Robbin's Well, at Leamington, and in the old well at Cheltenham, he estimated that there was only one grain in 60 gallons. The quantity is not at all proportionate to that of the muriate of soda in a spring; and at Llandridod, in Wales, it was found in water which contained none of this salt.

Very generally associated with iodine, is the congenerate substance bromine. Balard first discovered it in sea water, and subsequently it has been detected in several saline springs; it exists almost always as a hydro-bromate of magnesia.

"The largest quantity in the brine springs of England, was in those of Middlewich in Cheshire; Nantwich; Ashby de la Zouch, and Shirleywich. In the first, Dr. Daubeny found it to exist in the proportion of 1.337; in the second, of 0.903; in the third, 0.669; and in the last, 0.617. None was found in those springs at Cheltenham which contained iodine, whilst in the Pittville spring, the only one which does not contain it, every 6 gallons contain about a grain of bromine. A distinct trace was found in the water of Llandridod, which, as above mentioned, contains none of the muriate of soda." 28.

Besides the solid ingredients of mineral waters, there are several gazeous substances, which not unfrequently exist in very considerable quantities, and impart to them decided and characteristic properties; by far the most abundant of these is carbonic acid gas, which, when present in excess over the other ingredients, communicates a sparkling effervescence and an exhilarating sharpness to the waters; such are termed acidulous. The most celebrated are the Karlsbad, Pyrmont, Seltzer, Spa, Cheltenham, Tunbridge, &c. Carbonic acid escapes also in large quantities from the earth, in the form of gaseous springs, or solfataræ.

"Indeed Berthier has hazarded an opinion, which is perhaps the most correct view of the subject, that the gas which escapes with such profusion from many acidulated thermal wells, on their coming in contact with the external air, has never been in a state of combination with the water, but is an independent stream of carbonic acid discharged by the same fissure as the mineral water, and is far too copious to be absorbed by the latter. For, not to mention the immense evolutions of carbonic acid which are consequent upon every eruption of Vesuvius, and many other volcanoes, its quiet and peaceful exhalation is a phenomenon of very frequent occurrence in many different parts of the globe." 33.

The Poltersbrunn, at Franzensbad, in Bohemia, discharges great quantities of this gas; likewise the marsh behind the baths at Marienbad, the sulphur-hole near Ems, the Ragozi Well in Wurzburg, and several places in the basin of the Rhine. At Karlsbad, it issues in such quantities through the fissures of the great tufaceous vault on which the town is built, that the cellars of the houses in the lower part of the town are completely deserted. Every one has heard of the Grotto del Cane, near Naples; and of a similar nature are the Dunsthohle, near Pyrmont, the Puit de la Poule, near Neyrac, in the Vivarais, and probably the Gueno Upas, or poisoned valley in Java, and Alexander's Cave, near Tabriz, in Persia.

Another copious acid exhalation is that of sulphurous acid. It is a constant product of the lavas of Vesuvius and Ætna—of Stromboli and Bourbon; and Raffles describes a plain at Cheribon, in the N. E. of Java, 100 yards in diameter, which emits sulphurous vapours at every point. The sulphuric acid also, is frequently found; for example, in many of the grottoes in Italy—in a pool at Ætna—in the island of Milo—in the remarkable river which issues, highly impregnated with it, from the crater of Mount Ida, in Java, and in the Rio Vinagre, and adjoining hot springs in the Andes of Popayan, according to the statements of the celebrated Humboldt.

Muriatic acid vapours are emitted from volcanic localities, as from Vesuvius, Peak of Teneriffe, and Jorullo, in Mexico. The gaseous, non-acid ingredients of mineral waters are sulphuretted hydrogen, carburetted hydrogen, azote, hydrogen, and, lastly, oxygen and atmospheric air. When the first of these gases exist in predominating quantity, the springs are denominated—the sulphureous. In some cold springs, it is not an original ingredient, but the product of a partial decomposition of certain sulphates, arising from the contact of organic matters, such as particles of straw, chips of wood, &c. Besides the pure sulphuretted hydrogen, sulphur is met with in mineral waters as a hydro-sulphate, or, as it may be more appropriately termed, a hydro-thionate of lime or of soda. It is only by keeping this in view, that we can explain why some waters retain their sulphureous properties after boiling.

We have now glanced at almost all the ingredients, derived from the mineral kingdom, in cold as well as in thermal springs, and it only remains to allude to such as are of a vegetable or animal nature. The quantity of these is always very small, scarcely ever amounting to one part in 10,000 of water.

"In some cases, their presence is of easy explanation, being derived from beds of coal, petroleum, fossil vegetable or animal remains, or other bituminous substances situate in the route of a mineral water through its subterranean channels towards the surface. But that highly azotized principle, so frequent in many hot springs, is utterly inexplicable by this cause, as they are found to emerge from granite and crystalline schistous rocks, as in the Pyrenees, or from the ancient trachytes, products of former volcanic operations, as in central France, matrices utterly destitute of all vestiges of organic life." 42.

These vegeto-animal substances may be divided into three kinds:—1. Humus-extractive, which has been found by Berzelius in the Karlsbad, Königswart, and Kreuzbrunmen springs; and by Braconnot, in the thermal waters of Luxueil; this latter chemist called it Ulmin.

2. Resinous-extractive; it is closely allied in properties to mineral resin, and is most common in cold sulphureous and chalybeate springs; John detected it in the waters of Gleissen and Hermbstädt; and Paganini in those of several springs in Italy.

3. Animal-extractive, or Baregine; so denominated from its being found in considerable quantities in the hot sulphureous springs of Bareges in the Pyrenees. It is termed "Zoogen" by Gimbernat; "Theiothermin" by Monheim; and "Glairine" by Anglada. It is of a greyish-white colour, is unctuous and resinous; inodorous and without any sensible taste; is soluble in hot water, and when recovered by evaporation has a corneous aspect, and is semi-transparent; when burnt, it emits a strongly animal odour; by destructive distillation it yields carbonate of ammonia, azote, carburetted hydrogen and carbonic acid; it forms a soap with the caustic alcalis. One of its most characteristic qualities is, a very long exemption from putridity when exposed to all the necessary conditions of air, heat and moisture. On the whole we may regard it as allied more to mucus, than either to gelatine, or tannin. It is almost peculiar to thermal waters and very generally occurs in those of a very elevated temperature, as at Aix-la-Chapelle, Baden, Bareges, Wiesbaden, Teplitz, &c. Gimbernat found it in the hot vapours which rise from many springs from Vesuvius, and the Solfatara of Pozzuoli. Vauquelin and Thenard mention the occurrence of a 'matiere grasse' in the water of Provins near Paris. Much contrariety of opinion has existed, as to the probable origin of Baregine; that it cannot be derived from the percolation, and as it were, the lixiviation of fossil bones by the mineral waters, appears from the circumstance, that almost all the springs in which it is found, are known to issue from granite, mica-schist, trachyte, &c. which contain no traces of animal remains, nor even of the very elements of organic compounds.

"In a valuable memoir, Monheim has quoted a singular experiment of Dobereiner's, which, if not explaining, is extremely pertinent to the present question. Upon passing watery vapour over red hot coals, contained in an iron tube, in greater quantity than what could be decomposed, he obtained, besides carbonic acid, carbonic oxide, and carburetted hydrogen, a *gelatinous* substance, in such quantity as to close up the tube several times in succession: this substance was very soluble in water, and possessed many of the physical and chemical properties of tallow." 46.

Such is an outline of the most interesting ingredients of mineral waters; ample details on the exact and relative quantities, or the combinations, probable origin, and so forth, are contained in the elaborate work before us: to this we must refer the curious reader, who cannot fail to be alike instructed and amused by the many important facts brought under his notice. The short chapter on the "Constancy of the Impregnation of Mineral Waters" is particularly deserving of attention. How wonderful are the works of creation!—that the same fountain should pour out its gushing stream for centuries and thousands of centuries, ceaselessly, and for aught we know, without change or diminution, must astonish every one, and furnishes a boundless theme of speculation to the philosopher, as to the operations which are going on in the bowels of the earth, to feed its inward lakes with such enormous quantities of saline matter, and that too, uniformly, and in the same proportions and combinations; but this is a question which appertains rather to geology than to medicine, and we shall be satisfied with simply alluding to it. The next topic which engages our notice is that of the classification of mineral waters. On the first consideration, we are tempted to believe that this may be easily and satisfactorily done; but, in fact, it is not so, for although we are in the habit of speaking of acidulous, sulphureous, chalybeate, and saline springs, we must remember that the line of demarcation between each of these groupes is not nearly so distinct and obvious in Nature as in our books of chemistry, or rather that the line becomes frequently so faint as scarcely to be traced; and hence, at different periods, the same springs have been arranged as acidulous, as purging, and as chalybeate; the waters of Karlsbad are an example of this fluctuating uncertainty, and well illustrate that each of the views is in some measure correct, varying only according to the different bias of the enquirer, and the object of his pursuit.—The geologist looks at the vast tufaceous vault which encloses the boiling Sprudel, and which has been formed by the continued deposition of calcareous matter, and he is led to theorize on the important changes which have been, and may be effected by such springs on the physiognomy of the globe; the physician has a more limited scope, and he is satisfied with knowing that the waters are admirably tonic, in consequence, no doubt, of the iron which they contain; whereas the chemist tells us that they ought rather to be regarded as purging or saline, because the quantity of Glauber salt largely predominates over every other ingredient.

The same remarks are applicable to the waters of Bath, Wiesbaden, Teplitz, &c.; each of which has had various appellations, according to the varying medical and chemical philosophy of the age. Indeed it is not possible that the physician and the chemist can ever agree; and for this reason, that the therapeutic qualities of many springs are not in accordance with the predominating ingredient;—on this, as on many other occasions, Nature is repugnant to the wisdom of science. Dr. Black divided all mineral waters into six classes;—the thermal, acidulous, fossil alcaline, purging, chalybeate, and sulphureous. Saunders, in his excellent treatise, attempted to combine a chemical and a medical arrangement, as follows:—

"1. Simple cold, *e. g.* Malvern; 2. Simple thermal, *e. g.* Buxton; 3. Simple saline, *e. g.* Seidlitz, the Sea; 4. Highly carbonated alcaline, *e. g.* Seltzer; 5. Simple carbonated chalybeate, *e. g.* Tunbridge; 6. Hot carbonated chalybeate, *e. g.* Bath; 7. Highly carbonated chalybeate, *e. g.* Pyrmont; 8. Saline carbonated chalybeate, *e. g.* Cheltenham; 9. Hot saline highly carbonated chalybeate, *e. g.* Karlsbad; 10. Vitriolated chalybeate, *e. g.* Hartfell; 11. Cold sulphureous, *e. g.* Harrowgate; 12. Hot alkaline sulphureous, *e. g.* Bareges. This very complicated classification was succeeded by one of a totally opposite character, remarkable for its simplicity and extreme generality; it is that recognised by Murray, Alibert, and the majority of late writers on the subject. They are all comprised under the four classes of,—1. Carbonated; 2. Sulphureous; 3. Chalybeate; 4. Saline." 85.

The class of the saline waters in this last arrangement may be advantageously subdivided into three sections, according as the sulphates of soda or of magnesia, the muriate of soda, and the carbonate of soda, are predominant and characteristic;—we shall thus have the purging, the saline, or brine, and the alcaline waters. The mineral springs in Great Britain belong chiefly to the sulphureous, saline, and chalybeate classes; there are few alcaline or acidulous; and the number of hot or thermal waters is small, considering the great variety of geognostical distribution. In Scotland none of this latter class have hitherto been found; in England they form two distinct geographical groups; a northern in the county of Derby, including Buxton and Matlock—the temperature of these is very considerable; and a southern on the banks of the Avon, on the borders of Gloucester and Somerset. In this group we have the ancient waters of Bath, the hottest of the springs of England, and whose celebrity extends back to the age of the Romans; and the Bristol hot-well, the temperature of which is much inferior to that of Bath. It is worthy of notice, that the soil, out of which the Bath springs issue, emits copious bubbles of a gas, which was ascertained by Priestley to consist of 95 parts of azote, and 5 of carbonic acid. The only thermal water in Wales is that in the valley of Taafe, about six miles north of Cardiff in Glamorganshire; and Ireland possesses but one also, at Mallow, in Cork; its temperature is very inconsiderable. The chief sulphureous waters in Scotland are those of Strathpeffer in Ross, and of Moffatt in Dumfries; in England, those of Gilsland in Cumberland, Harrowgate in Yorkshire, Holbeck near Leeds, Leamington in Warwick, and in Wales, Llandridod in Radnorshire; and in Ireland, those of Lucan in the County of Dublin; several in Leitrim, and Swadlinbar in Cavan. The chief chalybeate waters of Great Britain are those of Hartfell near Moffat; Vicors Bridge, near Dollar in Fife; Peterhead; Bonnington near Edinburgh; Tunbridge, on the southern borders of Kent; Horley-Green, near Halifax, in Yorkshire; and one on the south coast of the Isle of Wight: and of Ireland are those of John's Well, in Kilkenny, Wexford Spa, Galway Spa, and Athlone. The saline springs are at Dunblane, near Stirling, [made classical by the labours of the late Dr. Murray,] Airthrey, Pitcaithly near Perth, Innerleithen on the Tweed, at Leamington, Cheltenham, Epsom, and Llandridod. The brine springs of England form two principal series; a smaller and more northerly in Durham; and a southern one, which is much more extensive, being distributed over the counties of Cheshire, at Middlewich, and Nantwich; Staffordshire at Shirleywich; and Worcestershire at Droitwich. In all of these the impregnation amounts almost to saturation; it is much less considerable in the springs of Kingswood near Bristol, of Bualt in Radnor, and of Ashby de la Zouch in Leicester. In Ireland the best known saline waters are at several places near Dublin—Cape Clear, Clonmell, Carrickmore, Antrim Spa, and Dromore. In reference to the division of alcaline springs, the only one in Britain, is that of Malvern; but the quantity of carbonate of soda is very small, and is associated with sulphate of soda and carbonate of lime. The principal purging waters are those of Scarborough and of Cheltenham. In the sandy vale at the latter place there is a great variety of springs, in some of which the sulphate of soda predominates, and in others the muriate; these two salts are however generally in nearly equal quantities, and are associated with the sulphate of magnesia, a little oxide of iron, and a slight sulphureous or carbonic acid impregnation.

It is not our intention to expatiate on the medical properties of the different classes of springs; a remark or two will suffice. Those who contend that the salutary effects of any spring may be inferred from its chemical composition are much at fault; for Murray has most ably shewn, that in our analyses, we often obtain a mere *product* and not the *educt* of the waters.

"The effects of a spring (says our author most justly) are often decidedly the reverse of what we should expect from its known chemical composition. Sometimes even, as has been more than once hinted in the foregoing part of this essay, common drinking springs contain more mineral matters than medicinal waters of great reputation; nay, in many of very great celebrity, chemistry has been able to detect so few active ingredients, that several have attributed their virtues solely to the great purity of their water. If this be the case with cold springs, it will certainly hold with increased force in regard to those possessing an elevated temperature. Warmth not only enables them to hold in solution a much larger quantity of active ingredients, but will give to very minute quantities a vastly increased activity; and finally renders soluble those deemed least so, as silica itself." 356.

The singularly beautiful speculations of Dr. Murray have certainly tended to throw considerable light on the medical history of mineral waters. In order to illustrate his views, let us take the Dunblane water; it contains sulphate of lime and muriate of soda; the former a wholly inert, and the latter in small quantities, not a very active substance; but if the constituents of these salts are interchanged, a muriate of lime and a sulphate of soda will be formed; and the quantity of the former ingredient will readily account for the efficacy of the spring in scrofulous complaints, and its cathartic powers will be referable to the Glauber salt;—indeed on no other supposition are these explicable; for the water contains no other ingredient to which we can ascribe these qualities.

Another consideration which ought to be borne in mind, and which should stimulate us to still more exact and rigidly-searching methods of analysis, is, that perhaps undiscovered principles or elementary substances exist in many of the most efficient springs;—and we are also to remember, that in many, perhaps in all, there are certain volatile ingredients which elude detection, even by the most delicate analysis. We know what a powerful influence in modifying the solid contents of a water is exerted by minute quantities of carbonic acid and of sulphuretted hydrogen; and we may fairly presume that similar or more powerful effects may be produced by other gases, which we have failed hitherto in discovering. No one suspected the presence of such an active ingredient as iodine in the saline waters of Sales in Piedmont, which had for ages before been celebrated for the cure of scrofula and bronchocele, till Angelina discovered it in 1820; and it is only within the last few years that the brine springs of Ashby de la Zouch in England, and of Kreuznach in the Palatinate, have been shewn by Danbery and Liebig to contain an unusually large proportion of the very potent principle—the hydrobromate of magnesia.

Little wonder, then, that the artificial mineral waters have so much fallen short of, in medical virtues, their natural prototypes. Formerly in the days of Nooth and Schweppe nothing was thought necessary in their preparation, except the appropriate employment of a simple compressing apparatus; but Struve of Dresden has by his unwearied and scientific labours brought the manufacture to infinitely greater precision and accuracy; he has succeeded in imitating perfectly some of the waters, as those of Karlsbad, but has failed in other attempts; thus his Ems water is much stronger and more violent than that of the natural springs. He is also obliged to admit that he has never yet been able to imitate satisfactorily those waters whose salutary efficacy does not correspond with the apparent insignificance of their mineral impregnation.

There was formerly a manufactory of artificial waters in London by Schweppe; at Geneva by Paul; at Ratisbon by Fries; and by Ziegler at Winterthur; but in the present day, the principal establishments are the *Brighton German Spa*, where the Karlsbad, Ems, Marienbad, Pyrmont, Spa, Seltzer, Saidschutz, &c. waters are prepared;—the *Tivoli* at Paris, in the Chaussée D'Antin; it is a large and excellent establishment, where the chief waters of France, Germany, and Italy may be had;—the *Oleggio* in Piedmont, instituted by Paganini ten years ago. It is delightfully situated on the summit of a hill, near the road over the Simplon, about four miles from the Lago Maggiore, and is much resorted to; a botanical garden, coffee-rooms, library and theatre, add much to its attractions;—the manu-

D 2

factory of the Karlsbad, at Stockholm, under the direction of Berzelius; and lastly, the establishments which have been set on foot by Struve himself at Dresden, Leipzig, Berlin, Konigsberg, Warsaw, and Moscow. Our readers are probably aware that almost all mineral waters undergo changes, when kept, or transported from their native springs; this is especially the case with the acidulous and chalybeate waters; the fixed air escapes, and the iron of the second class is precipitated in the state of oxide; different methods have been suggested to obviate the precipitation; we shall mention two.

"The first was proposed by Professor Steinmann of Prague (Hesperus for August 1821). It is founded upon the idea that the hydrated oxide of iron, which falls to the bottom of the most accurately corked bottles, might be separated by the tannin and gallic acid contained in the cork, as this is often coloured black at its point of contact with the water. He therefore advised the corks to be previously boiled, in order to extract these two principles, and found that, in bottles closed with boiled corks, the oxide of iron was not separated, after having stood for a considerable time, but amounted, by analysis of the water, to the original quantity of water contained in the spring.

The other method, originally suggested by Klaproth, and lately revived by Link (Hufeland's Journal, lxiv. st. 5, p. 3), consists in merely driving an iron nail through the cork. It has been even alleged that this increases the amount of iron held in solution by the water." 368.

Our time and space permit us to allude to only one subject more, and we have chosen that of "*Mineralised Mud Baths.*" Men are noted for torturing their brains to discover new pleasures, and strange and novel remedies; not satisfied with the wholesome and mundifying application of steam and water to their bodies, they have thought fit, on some occasions, to dip themselves in mud and mire, and have vehemently lauded the practice, as of singular efficacy. We had often heard of the native Hindoos piously killing their aged parents by seating them on the banks of the Ganges, at low water, till the mud and current gradually choked them; and if this did not happen quickly enough, by forcibly cramming their mouths, and closing up their eyes with the sacred mire; and we read that at Sacker in the Crimea, the practice of mud-bathing is in high repute among the sages of Tartary. As the account of it is interesting, we shall transcribe it. Sacker is a salt-lake, the evaporation of which in the hot Summer months, affords the deposit which is used :—

"Patients flock thither from all parts of the Crimea, and the cure lasts from eight to thirty days, and is conducted by the Tartar priests. The following is the method of using it detailed by Lang. Early in the morning a pit is dug, where the mud is freest from hard bodies and saline crystals. In this the patient is laid about noon, and covered up to the neck with the mineral mud. He is protected from the sun's rays by a parasol or cloth. He remains from two to three hours in this position, during which time the mud is renewed twice or thrice. His thirst is quenched by wine and water, or quass. After the bath, the patient is laid upon a straw-mat, and his whole body washed with the salt water of the adjoining lake. This process is considered by the natives a sovereign remedy for chronic gout and rheumatism, abdominal obstructions, glandular swellings, chronic cutaneous eruptions, and even intermittent fevers." 410.

But more learned men than Tartar priests have recommended the application of various muds to the body; Pliny speaks highly of it; and Galen notices with praise the use of the mud of the Nile; nor are we to deride it as altogether nugatory. Many remedies may be more pleasant, it is true; but it is quite possible, that the deposits from hot springs in particular, may exert considerable stimulating effects on the skin, especially when a douche of mineral water is afterwards used as a cleansing bath, and frictions are briskly employed; and that therefore this may be of occasional use in old gouts and rheumatisms, some chronic cutaneous diseases, palsies, scrofula, &c. The composition of mineralised mud is very various; most of the ingredients of mineral waters are found in it; the gaseous matters may have escaped, but their place is supplied by others, as carburetted hydrogen arising from a partial fermentation, or from the chemical re-action of the materials; and in addition to these a large proportion of organic matters is often present. When it consists of the simple deposit of any water, Duchanoy termed it "mare," and "bone" when the deposit was mixed with earthy matters; the Italians designate both kinds by the word "lutatura;" but we may conveniently distinguish three varieties:—1. The simple deposit of mineral waters highly charged with solid metallic and saline particles;—2. A mixed deposit of these particles, and of organic, chiefly vegetable matters, derived from the soil, and of gases arising from the fermentation; such are the mud-baths of Marienbad, and Franzenbad; and 3. Where, besides the ingredients hitherto mentioned, there is superadded a quantity of gelatinous azotised matter. This is found exclusively in thermal waters, which emit azotic gas, and which are generally sulphureous.

Osann has recognised six different kinds of mineral mud-baths—the *Sulphureous;* they are obtained from hot and cold sulphureous springs; have a peculiarly unctuous feel and a penetrating brimstone smell; the most noted are those of St. Amand, in Belgium; Eilsen, Gunthersbad, and Baden, in Germany; and of Acqui, in Piedmont; and of Abano, near Padua.

"This last has been celebrated since the times of Martial, Ammianus Marcellinus, Claudian, and Pliny. It is prepared by mixing fine clay and sand, which is placed in a pit or wooden trough, over which the thermal water is allowed to flow for some months. After having been used for the patients it is spread as manure over the fields. The mode of employing it is to spread a linen cloth with a layer of mud three or four inches thick, in which the whole body of the patient, or merely the affected part, is enveloped, and which is renewed as soon as the temperature falls." 406.

2. The *Carbonated,* which consists of the deposit of ferruginous mineral waters, mixed with vegetable earth, peat-bog, moss, &c.—they have a bituminous and slightly sulphureous smell; such are the mud-baths of Marienbad, Gleissen, Muskau, in Germany; and of Pyrmont and Audinac, in France.

3. The *Ferruginous,* resembling the carbonated, but containing more iron; and being thereby more astringent; the best known are at Loka, in Sweden; Medivi, in East Gothland; and at Piestan, in Hungary.

4. *Saline,* which are the product of evaporation of salt springs and lakes. Besides the one at Sacker in the Crimea, there are baths of this description at Elmen in Magdeburg, Ischl in Salzburg, and at Bourbon l'Archambault, and Bourbonne les Bains, in France.

5. The *Earthy.* This consists of brownish, soapy, sinter-like matter deposited by hot and cold springs, rich in carbonated earths, or of the frothy matter obtained by boiling many springs (the Badeschaum of the Germans.) Such are the baths at Schlangenbad, Wiesbaden, Hofgeismar, and Ussat; and—

6. Lastly the *Gelatinous*. The mud of these baths is a product of thermal water only, as we have stated before, and was known, as a remedial application, to Pliny, "muscus, qui in aqua fuerit, podagris illitur prodest." At Gastein, Baden, Neris and Dax, this ulva thermalis, as it has been called, is used in scrofulous sores, running tetters, &c.

We must now close our remarks on Dr. Gairdner's work, although a large portion of it has necessarily been left unreviewed; the chapters on the geography, topography, and geognosy of mineral springs, and on their probable origin, are of great interest, and well deserve the perusal of all lovers of natural history. As a whole, we regard it as a production of very high merit and eminent utility; the research which it displays is truly astonishing, and the judgment in selecting, arranging, and combining facts, from such varied sources, stamps the author as a most promising votary of science. He is young, but already has done much, and the horoscope of his scientific nativity augurs abundant success. We learn that he has been selected by the Hudson's Bay Company to go towards the Colombia River and the rocky mountains, on the Pacific side of North America, for the purpose of scientific research; his distinguished knowledge of botany and of geology admirably fits him for this enterprise, and leads us to anticipate much interesting information from his labours. We wish him every success, and assure him that it will afford us great pleasure, if we are again called upon to introduce him to the public as author of a work equal in talent to his Essay on Mineral and Thermal Springs.

